# Biomarkers of inflammation and endothelial dysfunction as predictors of pulse pressure and incident hypertension in type 1 diabetes: a 20 year life-course study in an inception cohort

**DOI:** 10.1007/s00125-017-4470-5

**Published:** 2017-11-03

**Authors:** Isabel Ferreira, Peter Hovind, Casper G. Schalkwijk, Hans-Henrik Parving, Coen D. A. Stehouwer, Peter Rossing

**Affiliations:** 1 Division of Epidemiology & Biostatistics, School of Public Health, Faculty of Medicine, The University of Queensland, Herston Campus – Public Health Building, Herston Rd, Brisbane, 4006 Australia; 2 Steno Diabetes Centre, Gentofte, Denmark; 3 Department of Clinical Physiology and Nuclear Medicine, Rigshospitalet, Glostrup, Denmark; 4 Department of Internal Medicine, Maastricht University Medical Centre (MUMC+), Peter Debyelaan 25, 6229HX Maastricht, the Netherlands; 5 CARIM School for Cardiovascular Diseases, MUMC+, Maastricht, the Netherlands; 6 Department of Medical Endocrinology, Rigshospitalet, University of Copenhagen, Copenhagen, Denmark; 7 Faculty of Health, University of Copenhagen, Copenhagen, Denmark; 8 HEALTH, Aarhus University, Aarhus, Denmark

**Keywords:** Arterial stiffness, CRP, Endothelial dysfunction, Hypertension, Inflammation, Longitudinal study, Pulse pressure, sE-selectin, sICAM-1, sVCAM-1, Type 1 diabetes

## Abstract

**Aims/hypothesis:**

Vascular inflammation and endothelial dysfunction are thought to contribute to arterial stiffening and hypertension. This study aims to test this hypothesis with longitudinal data in the context of type 1 diabetes.

**Methods:**

We investigated, in an inception cohort of 277 individuals with type 1 diabetes, the course, tracking and temporal inter-relationships of BP, specifically pulse pressure (a marker of arterial stiffening) and hypertension, and the following biomarkers of systemic and vascular inflammation/endothelial dysfunction: C-reactive protein (CRP), soluble intracellular adhesion molecule-1 (sICAM-1), soluble vascular cellular adhesion molecule-1 (sVCAM-1) and soluble E-selectin (sE-selectin). These biomarkers and other risk factors were measured at baseline and repeatedly up to 20 years after the onset of type 1 diabetes. Data were analysed with generalised estimating equations including adjustments for age, sex, smoking status, BMI, HbA_1c_, serum creatinine, total cholesterol, urinary AER, insulin treatment dose and mean arterial pressure.

**Results:**

Increases were noted in all biomarkers except sE-selectin, which decreased over time. Levels differed from baseline at 2–4 years and preceded the increase in pulse pressure, which occurred at 8–10 years after the onset of type 1 diabetes. Higher levels of sICAM-1 and sVCAM-1, but not CRP or sE-selectin, at baseline and throughout the 20 year follow-up, were significantly associated with higher (changes in) pulse pressure at subsequent time points. Higher levels of sVCAM-1 at baseline and during follow-up were also significantly associated with the prevalence (OR 3.60 [95% CI 1.36, 9.53] and OR 2.28 [1.03, 5.25], respectively) and incidence (OR 2.89 [1.08, 7.75] and OR 3.06 [1.01, 9.26], respectively) of hypertension. We also investigated the longitudinal associations between BP or hypertension as determinants of subsequent (changes in) levels of CRP, sICAM-1, sVCAM-1 and sE-selectin, but did not find evidence to support a reverse causality hypothesis.

**Conclusions/interpretation:**

These findings support the involvement of vascular endothelial dysfunction and inflammation in the development of premature arterial stiffening and hypertension in type 1 diabetes.

**Electronic supplementary material:**

The online version of this article (10.1007/s00125-017-4470-5) contains peer-reviewed but unedited supplementary material, which is available to authorised users.

## Introduction

Individuals with type 1 diabetes are characterised by accelerated arterial ageing [1, 2], a mechanism that increases the risk for cardiovascular disease (CVD) [2–4]. This has been illustrated by a steeper positive association between age and pulse pressure, a marker of arterial stiffness [5, 6], in individuals with type 1 diabetes than in their peers without the disease [1]. This is also supported by many studies demonstrating greater arterial stiffness in diabetes, as ascertained by measures of arterial pulse wave velocity or local estimates such as distensibility coefficients of the carotid and others arteries [3]. Importantly, in individuals with type 1 diabetes, higher pulse pressure (pulsatile load) has been associated with incident CVD independently of mean arterial pressure (MAP; steady load) [2].

The pathobiological mechanisms underlying the increases in pulse pressure in individuals with diabetes are, however, not clear. Diabetes is characterised by systemic and vascular inflammation and endothelial dysfunction [7–9], mechanisms that may link type 1 diabetes to increased pulse pressure [3]. Indeed, in recent years, inflammation and endothelial dysfunction, as expressed by levels of C-reactive protein (CRP) and cellular adhesion molecules (CAMs), have been proposed as putative determinants of arterial stiffness/pulse pressure and hypertension [10–12]. Epidemiological evidence to support this hypothesis remains weak, however, mainly due to limitations in study design. In addition, this has never been examined in the context of type 1 diabetes.

We have therefore investigated the longitudinal course of BP and markers of systemic and vascular inflammation and endothelial dysfunction (CRP, soluble intracellular adhesion molecule-1 [sICAM-1], soluble vascular cellular adhesion molecule-1 [sVCAM-1], and soluble E-selectin [sE-selectin]) and their temporal inter-relationships, in a cohort of individuals with type 1 diabetes who were followed over 20 years, since the onset of disease.

## Methods

### Study population and design

All individuals newly diagnosed with type 1 diabetes, consecutively admitted to the Steno Diabetes Centre between 1 September 1979 and 31 August 1984, were included in an inception cohort (*n* = 286), which has been described in detail previously [13, 14].

All participants attended the outpatient clinic every 3–4 months as part of their routine evaluation throughout a total follow-up period of ~20 years (median 18 years; range 1.1–21.5 years) up until 31 December 2000. These routine evaluations included measures of BP, HbA_1c_, BMI, serum cholesterol and creatinine, and urinary AER as described in detail elsewhere [13–15]. Throughout the years, participants were treated by diabetologists and nurses according to contemporaneous principles and guidelines [14, 16]. No specific intervention was carried out.

In line with previous reports in this cohort, we excluded from the analyses seven individuals with serious mental illness and two with microalbuminuria at onset of disease [13–15]. Therefore, the present study reports on analyses conducted using all clinical data obtained from 277 individuals over the course of follow-up. Of these individuals, 29 died and 19 moved out of care at the Steno Diabetes Centre at different follow-up times. The exact number of individuals analysed per time interval and the total number of observations made are summarised in electronic supplementary material (ESM) Table 1.

The local ethics committee (Copenhagen County, Denmark) approved the study and all individuals gave written informed consent for their participation in the study.

### Biomarkers of low-grade inflammation and endothelial dysfunction

All biomarkers were measured recently in stored blood samples that had been collected throughout the whole follow-up period. For each individual, samples spaced approximately 2 years apart were selected for these assessments. CRP was measured with a high-sensitivity in-house ELISA with rabbit anti-CRP (Dako, Copenhagen, Denmark) as a catching and tagging antibody as described previously, with intra- and inter-assay CV of 3.8% and 4.7%, respectively [8]. sVCAM-1, sICAM-1 and sE-selectin were measured in duplicate using commercially available ELISA kits (Diaclone, Besançon, France); the intra- and inter-assay CV was, respectively, 1.8% and 4.2% for sICAM-1, 1.1% and 3.1% for sVCAM-1, and 4.2% and 8.5% for sE-selectin.

### BP, hypertension and antihypertensive treatment

BP was measured at least once yearly with a standard sphygmomanometer and an appropriate cuff size. The measurements were performed, with participants in the sitting position after 10 min of rest, by attending physicians and trained nurses who were instructed to obtain two measurements. The average of these was reported in the participants’ files and used in the analyses. Because the recorded BP of patients receiving antihypertensive treatment is lower than the inherent untreated level, without appropriate correction, one is likely to obtain underestimates of the effects of potential determinants of BP [17]. Therefore, the recorded systolic BP (SBP) and diastolic BP (DBP) values under treatment were increased according to the algorithm proposed by Wu et al [18]. This method of adjustment is widely used in life-course analyses of BP [19] and has been shown to be effective at reducing the potentially distorting influence of antihypertensive treatment in studies examining its determinants [17]. Pulse pressure was then calculated as SBP – DBP and MAP as [(2 × DBP) + SBP] / 3.

In this cohort, criteria for the diagnosis of hypertension (triggering initiation of antihypertensive treatment) changed over the follow-up period: up until 1995 the WHO criterion (≥160/95 mmHg) was used, and the ADA criterion (≥140/90 mmHg) thereafter [20]. Because ~80% of all participants’ time in the study occurred while the WHO criteria were in use, their hypertension status thus mostly reflects these criteria. Throughout the years, an increasing range of antihypertensive drugs became available. Accordingly, before 1991, selective β-blockers, diuretics and vasodilators were used in this cohort. After 1991, β-blockers, diuretics, calcium channel blockers (mainly dihydropyridines) and ACE inhibitors (the predominant type of drug prescribed) were used. In addition, from 1994 onwards ACE inhibitors started to be used for the prevention of diabetic nephropathy [21] and, since then, patients who progressed to persistent microalbuminuria were treated with these drugs, regardless of their hypertensive status. Angiotensin II receptor blockers were used if ACE inhibitors were not tolerated (in seven patients only).

### Data organisation and statistical analyses

All analyses were carried out using STATA software package, version 14.0 (StataCorp, College Station, TX, USA).

All clinical data obtained from 6 months after the onset of diabetes (i.e. after initial stabilisation of blood glucose levels) up to 21.5 years thereafter (the maximum follow-up duration) were aggregated within 2 year time intervals. The number of readings available on the participants’ records varied per variable within each 2 year time interval (e.g. only one for the key determinants [biomarkers], only two [for BP] or more than two [body weight, HbA_1c_]); whenever more than one reading was available, median values were calculated when aggregating the data.

#### Missing data

Over the course of the follow-up period, different time-dependent variables were missing; sometimes this was transitory (if the variable was not measured or was measured but not noted in a participant’s file at intermittent time points) and sometimes data became permanently missing after a participant dropped out or died. We used the two-fold Fully Conditional Specification algorithm method for multiple imputation of longitudinal records of routinely collected clinical variables to impute missing data [22]. In brief, missing values at a given time point (*t*) were imputed conditional on all information available at the same and immediately adjacent *t.* We used a window of *t* ± 4 years and specified study entry and exit times for each participant so that imputations were not done outside these (i.e. after maximal follow-up date or death), which would assume an immortal cohort. Instead, in our study the target of inference was the mortal cohort (where only the surviving participants were included at each wave), hence the method chosen to impute the missing data [23]. Twenty imputed datasets were generated and all results reported are those from pooled analyses across all datasets.

#### Longitudinal analyses

All data were analysed by using linear (for continuous outcomes) or logistic (for dichotomous outcomes) generalised estimating equations (GEEs) to account, when appropriate, for the correlation and the unequal number of repeated observations taken in the same individuals over time [24]. Dichotomous outcomes were hypertension prevalence (proportion with hypertension at a given *t* irrespective of prior status) or incidence (proportion with a first diagnosis of hypertension at a given *t* among all those free of hypertension up to that *t*). All GEE models were fit using an exchangeable correlation structure, except for the models with incident hypertension as the main outcome where an independent correlation structure was used instead (analogous to a discrete time hazard model).

To investigate the mean yearly rates of change in BP and biomarkers, and the prevalence of hypertension at each *t*, we first examined the relationship of these variables with time. The exact (geometric) means and respective 95% CIs of biomarkers and BP and the prevalence of hypertension (in %) at each *t* were obtained by modelling time as a categorical variable (displayed graphically).

To analyse tracking of biomarkers and BP over the 20 year period, we used a model in which the initial value of each of these variables (i.e. at 0–2 years [*t*_0–2_]) was regressed on the entire subsequent levels of the same variable during follow-up (i.e. *t*_2–4_ to *t*_18–20_, tracking GEE model; see ESM Fig. 1a) [25]. Time-specific *z* scores of each variable were used in these analyses so that the tracking coefficients obtained could be interpreted as longitudinal correlation coefficients that range between 0 (no correlation) and 1 (perfect correlation).

We then used three complementary analytical models to examine the associations between biomarkers (main determinants) and BP (main outcomes), all modelling the levels of main determinants at time intervals prior to those of the outcomes, to minimise the possibility of overlap of the determinants and outcomes assessment times and enable inferences of causality. First, we examined the extent to which individual differences in biomarkers at baseline predicted individual differences in BP and the prevalence and incidence of hypertension over the entire follow-up period (baseline GEE model; ESM Fig. 1b). Second, we considered the time-varying nature of the determinants and the outcomes and examined their longitudinal associations with the use of a time-lagged GEE model (ESM Fig. 1c). With this model, we addressed the question of whether inter-individual differences in biomarkers at one *t* predicted subsequent inter-individual differences in BP (i.e. at *t* + 2 years). Finally, we investigated the extent to which inter-individual differences in main determinants at *t* predicted subsequent intra-individual changes in outcomes (i.e. between *t* + 2 and *t* + 4) using a time-lagged (changes) GEE model (ESM Fig. 1d). Time-lagged and changes models, as described above, were also used to examine the possibility of reverse causality, by considering BP or hypertension as determinants and biomarkers as outcomes.

All analytical models were first adjusted for time, and age at onset of diabetes, sex and smoking history (as time-independent covariates) and, second, for HbA_1c_, BMI, total cholesterol, urinary AER, serum creatinine and daily insulin dose (as time-dependent covariates). In analyses with pulse pressure as main outcome, we additionally adjusted for MAP to ascertain if any observed associations could be attributed to arterial stiffness independently of peripheral resistance [5]. Biomarkers were log_e_ transformed because of their right-skewed distribution. In analyses with these variables as main determinants of BP, longitudinal linear regression coefficients or ORs are therefore expressed as differences in BP (in mmHg) or odds of hypertension per doubling of each biomarker. In analyses of BP variables as main determinants of biomarkers (reverse causality models), longitudinal linear regression coefficients are expressed as per cent difference in biomarker per 10 mmHg increase in BP variable.

## Results

Characteristics of the study participants at baseline are shown in Table 1.

**Table 1:** Baseline characteristics of the study population

Characteristic	Measurement
Women, %	41.2
Age, years	27.4 ± 13.8
Smoking history^a^	
Never smoked, %	33.2
Ex smoker, %	21.3
Current smoker, %	45.5
Height, cm	171.6 ± 12.5
Weight, kg	64.1 ± 13.2
BMI, kg/m^2^	21.5 ± 2.6
HbA_1c_, %	8.1 ± 1.5
HbA_1c_, mmol/mol	65.0 ± 7.1
Insulin use, U/day	27.9 ± 11.7
Total cholesterol, mmol/l	5.14 ± 1.92
Serum creatinine, μmol/l	78.5 ± 16.7
Estimated GFR^b^, ml min^−1^ (1.73 m)^−2^	104.8 ± 23.1
Urinary AER, mg/24 h	9.8 (8.6, 11.1)
BP, mmHg	
SBP	121.8 ± 18.6
DBP	74.8 ± 11.3
MAP	90.4 ± 12.6
Pulse pressure	47.0 ± 13.7
Hypertension, %	1.1
CRP, nmol/l	5.71 (4.86, 6.76)
sICAM-1, ng/ml	639 (615, 663)
sVCAM-1, ng/ml	1189 (1148, 1230)
sE-selectin, ng/ml	142 (132, 152)

Data are shown as %, means ± SD or geometric means (95% CI); *n* = 277

^a^Includes 12 individuals who have smoked only during a portion of the follow-up

^b^Estimated according to the Chronic Kidney Disease Epidemiology Collaboration equation

### Longitudinal development of BP, hypertension and biomarkers

**Fig. 1:**
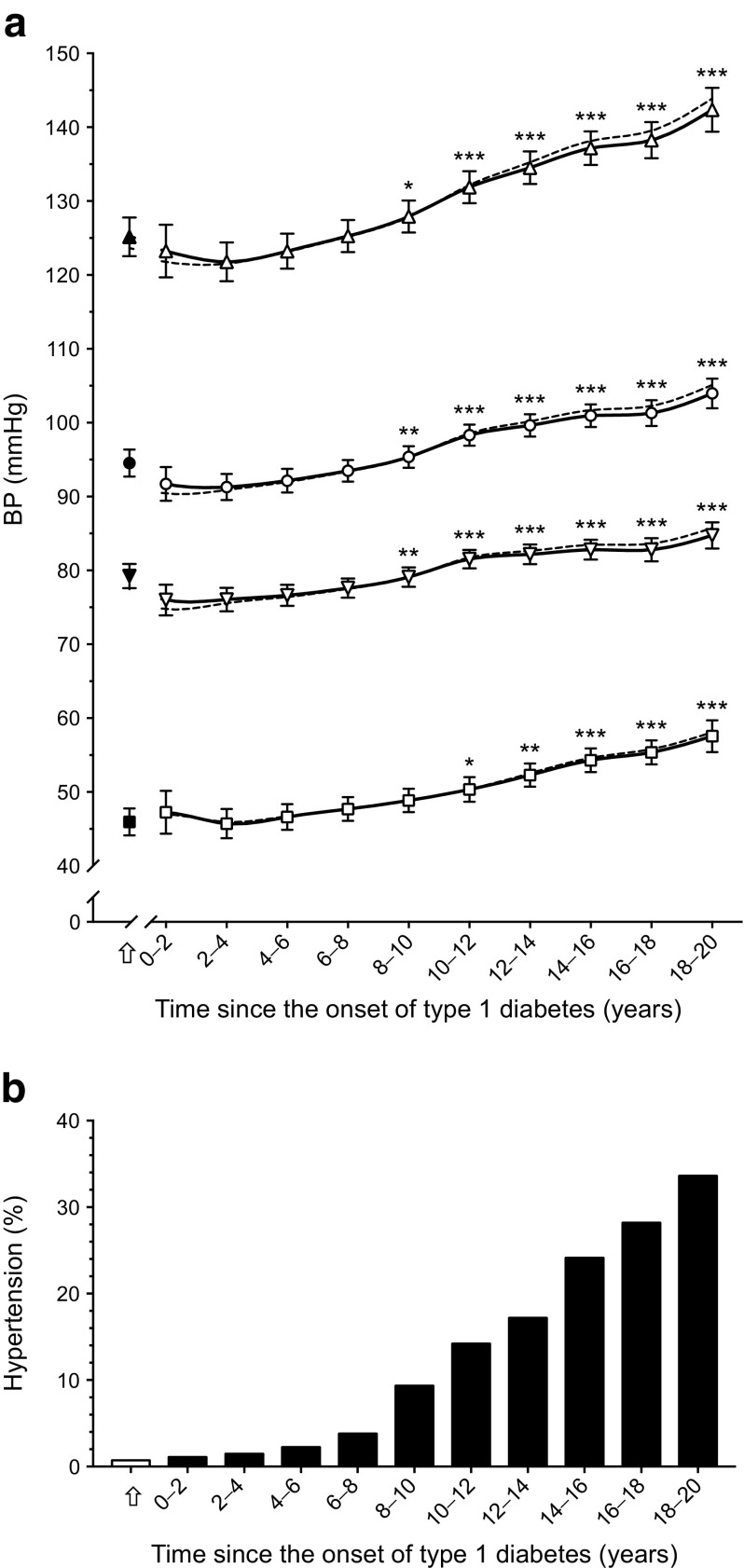
(**a**) Twenty-year time course of SBP (triangles), DBP (inverted triangles), MAP (circles) and pulse pressure (squares). Data show means and 95% CIs, adjusted for time-independent and time-dependent covariates; dashed lines represent unadjusted levels of BP. BP levels at disease onset (indicated by arrow) are shown for descriptive purposes only. (**b**) Estimated prevalence of hypertension at each time point. *p < 0.05, **p < 0.01 and ***p < 0.001 vs 0–2 years (baseline)

BP increased significantly over the 20 year longitudinal period (Fig. 1a). When a linear relationship with time was assumed, and after adjustments for all time-independent and time-dependent covariates, the average increase per year was 1.21 mmHg (95% CI 1.02, 1.40) for SBP, 0.53 mmHg (0.43, 0.64) for DBP, 0.76 mmHg (0.63, 0.89) for MAP and 0.67 mmHg (0.53, 0.80) for pulse pressure (all *p* < 0.001). The estimated prevalence of hypertension over the 20 year follow-up is shown in Fig. 1b.

**Fig. 2:**
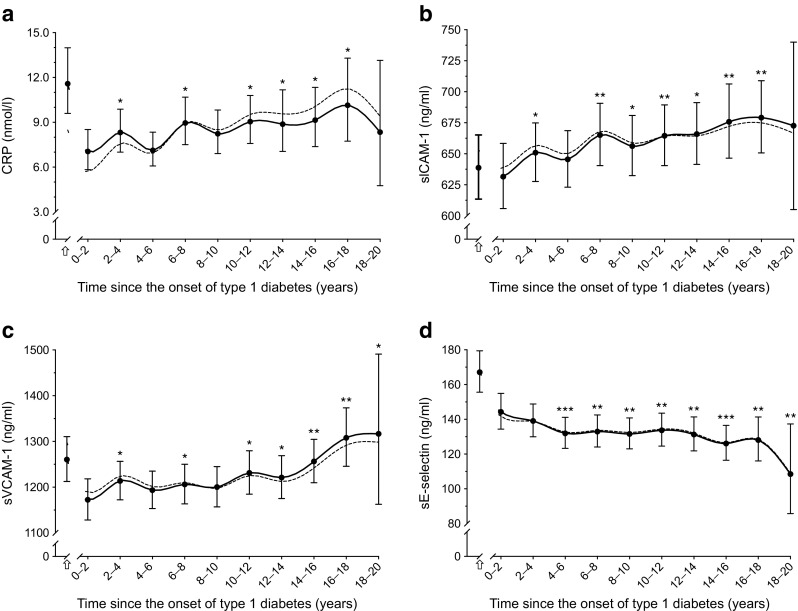
Twenty-year time course of CRP (**a**), sICAM-1 (**b**), sVCAM-1 (**c**) and sE-selectin (**d**). Data show geometric means and 95% CIs, adjusted for time-independent and time-dependent covariates; dashed lines represent unadjusted levels of biomarkers. The levels of biomarkers at disease onset (indicated by arrow) are shown for descriptive purposes only. *p < 0.05, **p < 0.01 and ***p < 0.001 vs 0–2 years (baseline)

Markers of low-grade inflammation and endothelial dysfunction also changed significantly over time (Fig. 2a–d). When a linear relationship with time was assumed, and after adjustments for all covariates, there was a yearly increase of 1.7% (95% CI 0.2, 3.2) for CRP, 0.3% (0.1, 0.7) for sICAM-1 and 0.6% (0.2, 0.9) for sVCAM-1, whereas sE-selectin decreased by 0.9% (−1.4, −0.3) each year (all *p* < 0.05).

The increases in levels of biomarkers (except sE-selectin) were observed early; levels already differed from baseline at 2–4 years after onset of disease and preceded the increases in BP, which differed significantly from baseline at 8–10 years after the onset of type 1 diabetes (Figs 1 and 2).

### Tracking of BP and biomarkers

After adjustments for all time-independent and time-dependent covariates, tracking coefficients of BP over the 20 years of follow-up ranged between 0.26 (pulse pressure) and 0.36 (SBP and MAP) (Table 2). Except for CRP, tracking coefficients for the biomarkers were considerably higher (all ≥0.58) (Table 2).

**Table 2:** Tracking of BP and biomarkers of inflammation and endothelial dysfunction over the 20 years of follow-up

Variable	Model 1^a^	Model 2^b^
BP		
SBP	0.40 (0.29, 0.51)	0.36 (0.26, 0.47)
DBP	0.32 (0.19, 0.46)	0.30 (0.17, 0.42)
MAP	0.39 (0.26, 0.52)	0.36 (0.23, 0.48)
Pulse pressure	0.28 (0.17, 0.39)	0.26 (0.15, 0.36)
Biomarkers		
CRP	0.28 (0.18, 0.36)	0.26 (0.20, 0.33)
sICAM-1	0.60 (0.52, 0.67)	0.61 (0.57, 0.66)
sVCAM-1	0.58 (0.50, 0.66)	0.58 (0.53, 0.64)
sE-selectin	0.78 (0.71, 0.84)	0.79 (0.75, 0.82)

Data show 20 year tracking coefficients (95% CI); these are equivalent to longitudinal correlation coefficients with values that can vary between 0 (no correlation) and 1 (perfect correlation)

^a^Model 1, adjusted for sex, age at onset of type 1 diabetes, smoking status (time-independent covariates) and time

^b^Model 2, model 1 further adjusted for all other time-dependent covariates (i.e. BMI, HbA_1c_, total cholesterol, serum creatinine, urinary AER, insulin dose and MAP or biomarkers)

All tracking coefficients were significant at *p* < 0.001 level

### Longitudinal associations between biomarkers and pulse pressure

After adjustments for age, sex and smoking (model 1) and for the baseline levels of the other risk factors (model 2), higher levels of sICAM-1 and sVCAM-1, but not of CRP or sE-selectin, at baseline were significantly associated with higher pulse pressure during the 20 years of follow-up (baseline GEE model, Table 3). However, the association with sVCAM-1 was attenuated and no longer significant after additional adjustment for MAP (model 3).

**Table 3:** Longitudinal associations between biomarkers of inflammation/endothelial dysfunction and pulse pressure

GEE model/main determinant	Model 1^a^	Model 2^b^	Model 3^c^
Baseline			
CRP	−0.01 (−0.81, 0.80)	−0.04 (−0.86, 0.78)	−0.13 (−0.74, 0.49)
sICAM-1	3.22 (0.11, 6.33)*	3.19 (0.04, 6.35)*	2.97 (0.13, 5.80)*
sVCAM-1	3.76 (0.20, 7.33)*	3.84 (0.20, 7.49)*	1.76 (−1.33, 4.86)
sE-selectin	1.19 (−0.75, 3.13)	1.22 (−0.76, 3.21)	0.76 (−0.76, 2.27)
Time-lagged			
CRP	0.75 (0.18, 1.33)*	0.27 (−0.32, 0.86)	0.26 (−0.28, 0.80)
sICAM-1	3.93 (0.62, 7.25)*	3.51 (0.24, 6.79)*	3.15 (0.44, 5.87)*
sVCAM-1	5.13 (1.86, 8.40)**	5.06 (1.86, 8.26)**	3.23 (0.49, 5.98)*
sE-selectin	0.95 (−0.70, 2.61)	0.99 (−0.71, 2.69)	0.92 (−0.43, 2.27)
Time-lagged (changes)			
CRP	0.38 (*−*0.09, 0.85)	0.13 (−0.37, 0.63)	0.18 (−0.32, 0.67)
sICAM-1	2.04 (−0.23, 4.31)	2.09 (−0.28, 4.47)	2.47 (0.16, 4.78)*
sVCAM-1	3.36 (0.85, 5.86)**	3.53 (0.93, 6.12)**	2.54 (0.12, 4.96)*
sE-selectin	0.70 (−0.35, 1.76)	0.63 (−0.54, 1.81)	0.62 (−0.49, 1.73)

Data show longitudinal linear regression coefficients (95% CI), indicating difference (in baseline and time-lagged models) or increase (changes model) in blood pressure (in mmHg) per doubling in biomarker (*n* = 277)

To re-express these association estimates per 10% increase in biomarker multiply by log_e_(1.1) = 0.095 or ~0.1

^a^Model 1, adjusted for sex, age at onset of type 1 diabetes, smoking status (time-independent covariates) and time

^b^Model 2, model 1 further adjusted for BMI, HbA_1c_, total cholesterol, serum creatinine, urinary AER and insulin dose (time-dependent covariates, except in the baseline model where only their baseline levels were considered)

^c^Model 3, model 2 further adjusted for (changes in) MAP (time-dependent covariate)

*p < 0.05 and **p < 0.01

In analyses considering the time-varying nature of not only the outcomes (BP) but also the main exposures (biomarkers), and after adjustments for age, sex and smoking status (model 1), CRP, sICAM-1 and sVCAM-1 were all significantly associated with higher subsequent pulse pressure: 0.75 mmHg (95% CI 0.18, 1.33), 3.93 mmHg (0.62, 7.25) and 5.13 mmHg (1.86, 8.40) per doubling in each biomarker, respectively (time-lagged GEE model, Table 3). The adjustments for the time-varying covariates attenuated the association between CRP and pulse pressure markedly, to 0.27 mmHg (−0.32, 0.86), but did not importantly affect the associations between sICAM-1 or sVCAM-1 and pulse pressure (models 1 vs 2). After further adjustments for MAP (model 3), both sICAM-1 and sVCAM-1 remained significantly associated with subsequent pulse pressure, although the strength of the association with sVCAM-1 was attenuated more markedly: 3.15 mmHg (0.44, 5.87) and 3.23 mmHg (0.49, 5.98) per doubling in sICAM-1 and sVCAM-1, respectively.

After adjustments for all covariates considered, including changes in MAP (model 3), higher levels of sICAM-1 and sVCAM-1, but not of CRP or sE-selectin, also predicted subsequent changes in pulse pressure: 2.47 mmHg (0.16, 4.78) and 2.54 mmHg (0.12, 4.96) per doubling in sICAM-1 and sVCAM-1, respectively (time-lagged changes GEE model, Table 3).

The significant associations between CAMs and pulse pressure described above derived from distinct patterns of association; specifically, sVCAM-1 was positively associated with both SBP and DBP but more strongly so with SBP, whereas sICAM-1 was positively associated with SBP but inversely associated with DBP (ESM Table 2).

### Longitudinal associations between biomarkers and hypertension

Among the biomarkers investigated, and in all GEE models considered, sVCAM-1 was the only independent predictor of both the prevalence and the incidence of hypertension throughout the follow-up period (Table 4).

**Table 4:** Longitudinal associations between biomarkers of inflammation/endothelial dysfunction and hypertension

GEE model/main determinant	Prevalence of hypertension (*n* = 277)		Incidence of hypertension (*n* = 271)^a^	
	Model 1^b^	Model 2^c^	Model 1^b^	Model 2^c^
Baseline^a^				
CRP	1.12 (0.92, 1.36)	1.06 (0.87, 1.29)	1.14 (0.91, 1.42)	1.13 (0.90, 1.43)
sICAM-1	1.79 (0.78, 4.10)	1.77 (0.74, 4.25)	2.19 (0.87, 5.53)	2.26 (0.85, 5.80)
sVCAM-1	3.02 (1.25, 7.31)*	3.60 (1.36, 9.53)**	2.83 (1.10, 7.27)*	2.89 (1.08, 7.75)*
sE-selectin	1.65 (1.03, 2.65)*	1.44 (0.87, 2.38)	1.42 (0.85, 2.39)	1.37 (0.78, 2.39)
Time-lagged				
CRP	1.13 (0.98, 1.30)	1.00 (0.88, 1.15)	1.23 (0.95, 1.59)	1.21 (0.92, 1.59)
sICAM-1	2.21 (1.06, 4.60)*	1.90 (0.91, 4.00)	2.73 (0.96, 7.76)	2.89 (0.93, 9.01)
sVCAM-1	2.40 (1.16, 4.94)*	2.28 (1.03, 5.25)*	3.00 (1.06, 8.54)*	3.06 (1.01, 9.26)*
sE-selectin	1.14 (0.74, 1.77)	1.18 (0.78, 1.77)	1.66 (0.94, 2.93)	1.53 (0.82, 2.83)

ORs (95% CIs) are shown for prevalence of hypertension or for incidence of hypertension per doubling in biomarker

^a^Excludes three individuals with hypertension at baseline and three who died before the first follow-up measurement

^b^Model 1, adjusted for sex, age at the onset of type 1 diabetes, smoking status (time-independent covariates) and time;

^c^Model 2, model 1 further adjusted for BMI, HbA_1c_, total cholesterol, serum creatinine, urinary AER and insulin dose (time-dependent covariates, except in the baseline model where only their baseline levels were considered);

*p < 0.05 and **p < 0.01

### Reverse causality?

We also investigated the longitudinal associations between BP or hypertension as determinants of subsequent (changes in) levels of CRP, sICAM-1, sVCAM-1 and sE-selectin, but did not find evidence to support a reverse causality hypothesis (ESM Table 3).

### Additional analyses

Given that CRP and BP were associated with each other but seemingly not in a causal fashion, we examined an alternative hypothesis: that both CRP and BP were, instead, determined by (a) common factor(s). After adjustments for the time-independent and time-dependent covariates, we found that (changes in) BMI (per kg/m^2^) observed in the course of follow-up (mean rate of increase 0.12 kg/m^2^ per year [95% CI 0.10, 0.13], *p* < 0.001) was the single common determinant of subsequent pulse pressure (0.58 mmHg [0.26, 0.89] in time-lagged GEE model and 0.32 mmHg [0.09, 0.55] in change GEE model), prevalence of hypertension (OR 1.22 [1.14, 1.33]), incidence of hypertension (OR 1.10 [1.00, 1.21]) and subsequent levels of CRP (5.0% [1.5, 8.5] in time-lagged GEE model and 3.0% [0.1, 5.9] in time-lagged changes GEE model).

Additional analyses of whether the associations reported differed by age at onset, sex, (micro)albuminuria status or time revealed no consistent effect modification by any of these factors.

## Discussion

We examined the longitudinal course of BP and biomarkers of endothelial dysfunction and inflammation, and their inter-relationships, in a cohort of individuals with type 1 diabetes who had been followed for 20 years since the onset of disease. Our main findings were as follows: (1) increases in levels of CRP, sICAM-1 and sVCAM-1 occurred early in the course of disease and preceded the increases in BP; (2) all variables tracked considerably over time, particularly sICAM-1, sVCAM-1 and sE-selectin; (3) higher sICAM-1 and sVCAM-1, at baseline and during follow-up, predicted the subsequent (changes in) levels of pulse pressure; higher levels of sVCAM-1, at baseline and during follow-up, predicted the prevalence and incidence of hypertension; (4) notably, these associations were independent of age, sex, smoking history and other relevant risk factors and (5) we found no evidence for a reverse causation hypothesis (i.e. that higher levels of BP or hypertension determined subsequent increases in these biomarkers). The unique characteristics of this study were the repeated assessment of biomarkers, BP and important covariates over the natural course of disease during a period of 20 years. This allowed us to examine, for the first time with a truly longitudinal design, the temporal relationships between biomarkers and BP (notably, pulse pressure) in individuals with type 1 diabetes. As such, the present study provides the strongest evidence regarding the involvement (or lack thereof) of CRP, sICAM-1, sVCAM-1 and sE-selectin in the pathogenesis of elevated pulse pressure and of hypertension, over the course of disease in individuals with type 1 diabetes.

Tracking of BP and CRP levels over the 20 year follow-up period was moderate but was very high for sICAM-1, sVCAM-1 and sE-selectin. Interpretation of the tracking coefficients reported here requires consideration of a number of factors [25]. First, these coefficients reflect only the stability of one’s rank position vs peers over time. Second, the magnitude of these coefficients tends to decrease with the length of follow-up. Remarkably, the tracking coefficients of the BP variables were comparable with those reported in a 15 year longitudinal study among healthy individuals followed from adolescence to young adulthood [25]. To our knowledge, long-term tracking coefficients for the biomarkers examined herein have never been reported before. The very high tracking of both CAMs suggests that they can be used to identify, early in the disease, those who are likely to remain with adverse levels leading to related sequelae. Third, measurement error attenuates tracking coefficients, which may explain why these were stronger for the biomarkers than for BP. Indeed, throughout the years, BP was measured in the clinical setting as part of the routine follow-up care provided to patients by different attending physicians and nurses, whereas biomarkers were all assessed at the same laboratory at the same time, using the same methodology. Finally, tracking coefficients may also be affected by extraneous factors, although adjustments for both time-independent and time-dependent covariates only seemed to attenuate their magnitude slightly.

Epidemiological evidence to support the concept of arterial stiffness/widened pulse pressure and hypertension as a consequence of vascular/systemic inflammation has been controversial [11, 26–29]. Our findings are in agreement with previous studies showing that higher levels of CRP are related to hypertension and pulse pressure [26, 30–35]. However, after life-course adjustments for confounders, the associations with pulse pressure and hypertension were markedly attenuated and no longer significant, suggesting that these associations are not causal. These findings are supported by previous studies showing that the cross-sectional associations between CRP and hypertension, as well as pulse pressure [27] or aortic pulse wave velocity [29], disappeared after adjustment for life-course confounding or Mendelian randomisation tests of causality. Furthermore, although some prospective studies have shown associations between CRP and incident hypertension [36, 37], others have shown these to disappear after adjustment for BMI [38], suggesting that BMI is a common determinant of both pulse pressure/hypertension and CRP [11]. Our data supports this hypothesis. Increases in BMI and related BP in individuals with type 1 diabetes have been well documented as long-term consequences of intensified insulin treatment [39, 40]. In addition, increases in CRP, but not in CAMs, have been shown to depend on the degree of weight gain in insulin-treated patients with type 1 diabetes [41]. We [8, 42], and others [7], have also shown that BMI is the strongest correlate of CRP in these individuals.

Our findings suggest that, in contrast to CRP (and sE-selectin), both CAMs may causally underlie arterial stiffening. This contention holds inasmuch as arterial stiffness can be depicted by pulse pressure and may be too strong given that stroke volume (not measured) is also likely to explain a part of the variance in pulse pressure, particularly among young individuals. Our findings also suggest that sVCAM-1 seems to causally underlie the development of hypertension. This observation is in striking agreement with the only prospective study thus far that has examined CAMs and pro-inflammatory cytokines as predictors of incident hypertension among individuals with type 1 diabetes [43]. Indeed, in that study only the baseline levels of sVCAM-1 (but not sICAM-1, CRP, IL-6 and TNF-α) were associated with the 15 year cumulative incidence of hypertension. We extended these observations by examining not only associations with levels of biomarkers at baseline but also their changes during follow-up. Soluble CAMs are markers of vascular endothelial dysfunction/inflammation that may influence functional stiffening of large arteries through reduced availability of nitric oxide and/or increased activity of vasoconstrictors such as endothelin-1, both of which affect vascular smooth muscle cell tone [9]. In addition, endothelial dysfunction/inflammation may lead to smooth muscle cell proliferation and increased synthesis of structural proteins such as collagen within the vascular wall, leading to structural stiffening of large arteries [3, 9]. Previous studies on soluble CAMs and BP have produced contradictory findings but have been mostly cross-sectional and not focused on the pulsatile component of BP [44–50]. Appreciation of the BP curve as a summation of a steady component (MAP) and a pulsatile component (pulse pressure) has provided additional information in terms of CVD risk prediction to that traditionally obtained on the basis of elevated SBP and/or DBP alone [5].

We used brachial, not central, pulse pressure as a crude estimate of arterial stiffness, reflecting the data that was accessible from clinical records. The technology enabling non-invasive measurement of central pulse pressure and aortic pulse wave velocity, that would allow better characterisation of the aetiology of arterial stiffening, was not available during the 20 year period covered by the present study. Nevertheless, brachial pulse pressure still provides valuable risk prediction information: in a meta-analysis (not including individuals with type 1 diabetes), central pulse pressure tended to be more strongly associated with incident CVD and mortality than brachial pulse pressure but the added value of central pulse pressure in risk prediction was only marginal [51]; a similar pattern was observed in a large cohort study of individuals with type 1 diabetes [4].

There are some additional limitations to our study. Findings were confined to individuals with type 1 diabetes and therefore may not generalise to the background population. Measurement errors around BP measurements are likely to have led to an underestimation of the associations estimates reported in the present manuscript. We only measured CRP, sICAM-1, sVCAM-1 and sE-selectin, which reflect just a part of the complex and multifaceted process of arterial remodelling induced by endothelial dysfunction and inflammation [9]. Still, although the CAMs studied here may be produced by different cell types, changes in their plasma levels are widely considered to reflect altered endothelial production rates [9]. The biomarkers were measured on stored blood samples, which raises the question of whether the reported increases in their levels over time could in part reflect a decay of the proteins with storage time. We deem this unlikely because of the high long-term stability of concentrations of these proteins in stored serum [52]. Besides, while levels of CRP, sICAM-1 and sVCAM-1 increased, levels of sE-selectin decreased over time. In addition, despite the long storage time, our measurements could capture considerably higher levels of biomarkers at disease onset (excluded from the analyses) followed by decreases after blood-glucose stabilisation, with relatively steady increases (except for sE-selectin) thereafter (see Fig. 2).

In conclusion, in individuals with type 1 diabetes, increases in sICAM-1 and sVCAM-1 precede, and are associated with, subsequent increases in pulse pressure and hypertension throughout the course of the disease, supporting the involvement of endothelial dysfunction/inflammation in the development of premature arterial stiffening. The lack of support for a causal link between CRP and BP, and the observation that both derive from a common antecedent (BMI), suggests that weight gain should be monitored during treatment of individuals with type 1 diabetes. Targeting endothelial dysfunction/inflammation in the early stages of diabetes may slow down the accelerated arterial ageing characteristic of this disease and prevent related cardiovascular sequelae. Given that both CAMs tracked very highly, measuring their levels and changes soon after the onset of type 1 diabetes may enable identification of individuals at a high risk and who may need intensified/tailored treatment.

## Electronic supplementary material

ESM (PDF 209 kb)

## Abbreviations

CAM: Cellular adhesion molecule
CRP: C-reactive protein
CVD: Cardiovascular disease
DBP: Diastolic BP
GEE: Generalised estimating equation
MAP: Mean arterial pressure
SBP: Systolic BP
sE-selectin: Soluble E-selectin
sICAM-1: Soluble intracellular adhesion molecule-1
sVCAM-1: Soluble vascular cellular adhesion molecule-1
