# Supplementary material for: Biomarkers of inflammation and endothelial dysfunction as predictors of pulse pressure and incident hypertension in type 1 diabetes: a 20 year life-course study in an inception cohort
Source: Diabetologia. 2017 Nov 3;61(1):231–41. doi: 10.1007/s00125-017-4470-5 (PMC6448953; doi:10.1007/s00125-017-4470-5)
Supplement: Supplementary file 1 — (PDF 209 kb) [file 125_2017_4470_MOESM1_ESM.pdf]

Electronic supplementary material (ESM)

ESM Table 1 Number of participants, deaths and dropouts per aggregated time interval.

**Time interval (years)**	**0-2[a]**	**2-4**	**4-6**	**6-8**	**8-10**	**10-12**	**12-14**	**14-16**	**16-18**	**18-20**	**Total no. observations (cases)**
N	277	274	273	272	269	265	259	257	250[d]	*151[d]*	2547
Deaths[b]	3	1	1	3	4	6	2	2	3	4	(29)
Dropouts[c]	0	1	3	0	6	1	3	6	-	-	(19)

N, number of patients analysed at each follow-up time interval.

[a]Indicates baseline assessments excluding the first 6 months after onset of diabetes to allow for stabilisation of blood-glucose levels;

[b]Indicates number of patients who deceased during the time interval; all still contributed data up to the time of death; data missing thereafter was thus not imputed for these patients (inferences made for the 'mortal' cohort).

[c]Missing data from these patients were imputed (based on observed data) up to their potential maximum time in the study.

[d]The lower number of patients with data at the last time intervals (i.e. 250 and 151) is due to their later inclusion in the study (inclusion took place between September 1979 and August 1984; end of follow-up terminated in Dec 2000).

ESM Table 2. Longitudinal associations between markers of inflammation/endothelial dysfunction and systolic (SBP) or diastolic (DBP) blood pressure.

		SBP		DBP	
**GEE Model**	**Main determinants**	**Model 1[a]**	**Model 2[b]**	**Model 1[a]**	**Model 2[b]**
**Baseline**	**CRP**	0.31 (-1.04, 1.66)	0.14 (-1.24, 1.51)	0.31 (-0.48, 1.11)	0.17 (-0.63, 0.97)
	**sICAM-1**	2.39 (-3.30, 8.07)	2.66 (-3.09, 8.41)	-0.87 (-4.25, 2.51)	-0.57 (-3.90, 2.75)
	**sVCAM-1**	6.20 (0.35, 12.06)*	6.54 (0.64, 12.44)*	2.46 (-1.21, 6.13)	2.70 (-1.01, 6.41)
	**eSEL**	1.94 (-1.26, 5.14)	1.69 (-1.56, 4.95)	0.74 (-1.13, 2.61)	0.45 (-1.42, 2.32)
**Time-lagged**	**CRP**	1.25 (0.54, 1.96)**	0.18 (-0.52, 0.89)	0.48 (0.03, 0.93)*	-0.09 (-0.52, 0.34)
	**sICAM-1**	3.91 (-0.59, 8.40)	3.13 (-1.11, 7.38)	-0.05 (-2.43, 2.33)	-0.32 (-2.60, 1.96)
	**sVCAM-1**	6.10 (1.63, 10.58)**	6.21 (2.03, 10.40)**	0.91 (-1.44, 3.26)	1.10 (-1.14, 3.35)
	**eSEL**	0.11 (-2.61, 2.84)	0.39 (-2.21, 2.99)	-0.77 (-2.41, 0.87)	-0.57 (-2.03, 0.88)
**Time-lagged**	**CRP**	0.24 (-0.26, 0.74)	-0.04 (-0.59, 0.50)	0.16 (-0.22, 0.54)	-0.09 (-0.47, 0.29)
**(changes)**	**sICAM-1**	0.72 (-1.44, 2.89)	0.97 (-1.52, 3.47)	-0.28 (-1.94, 1.39)	-0.30 (-2.08, 1.48)
	**sVCAM-1**	2.63 (0.17, 5.09)*	3.21 (0.47, 5.97)*	0.66 (-1.14, 2.46)	0.78 (-1.09, 2.64)
	**eSEL**	0.58 (-0.48, 1.64)	0.44 (-0.85, 1.75)	0.09 (-0.78, 0.97)	-0.05 (-1.00, 0.89)

Data show longitudinal linear regression coefficients (95% CI), indicating difference in blood pressure (in mmHg) per doubling in biomarker
To re-express these association estimates per 10% increase in biomarker multiply by $\log_e(1.1)=0.095$ or ~0.1
[a] Model 1, adjusted for sex, age at onset of type 1 diabetes and smoking status (time-independent covariates) and time
[b] Model 2, model 1 further adjusted for BMI, HbA1c, total cholesterol, serum creatinine and urinary AER and insulin dose
**p*<0.05 and ***p*<0.01

ESM Table 3. Longitudinal associations between blood pressure or hypertension and biomarkers of inflammation/endothelial dysfunction *(**reverse causality hypothesis**).*

			Main outcomes			
**GEE Model**	**Main Determinants**	**Model**	**CRP**	**sICAM**	**sVCAM-1**	**sE-Selectin**
**Baseline**	***Pulse pressure***	1[a]	5.7 (-4.8, 17.4)	1.9 (-0.7, 4.4)	1.9 (-0.7, 4.5)	2.0 (-4.0, 8.5)
		2[b]	3.7 (-8.0, 17.0)	1.5 (-1.5, 4.6)	1.3 (-1.7, 4.3)	0.8 (-6.2, 8.4)
	SBP	1[a]	4.8 (-2.9, 13.2)	1.2 (-0.7, 3.2)	1.6 (-0.1, 3.3)	2.6 (-2.0, 7.3)
		2[b]	4.3 (-3.5, 12.7)	1.3 (-0.7, 3.3)	1.6 (-0.1, 3.3)	2.5 (-2.2, 7.4)
	DBP	1[a]	4.0 (-8.2, 17.6)	0.5 (-3.0, 4.1)	1.4 (-1.7, 4.6)	3.8 (-3.9, 12.2)
		2[b]	3.6 (-8.8; 17.6)	0.8 (-2.8, 4.4)	1.5 (-1.5, 4.7)	3.4 (-4.4, 11.8)
**Time-lagged**	***Pulse pressure***	1[a]	4.5 (-2.0, 11.3)	0.5 (-0.5, 1.5)	1.0 (-0.1, 2.1)	-0.1 (-1.9, 1.7)
		2[b]	1.5 (-4.7, 8.0)	0.5 (-0.6, 1.5)	0.6 (-0.4, 1.5)	1.1 (-1.1, 2.3)
	SBP	1[a]	4.9 (-0.1, 10.2)	0.3 (-0.5, 1.1)	0.9 (-0.0, 1.8)	-0.6 (-2.1, 0.9)
		2[b]	1.6 (-3.2, 6.4)	0.3 (-0.5, 1.1)	0.9 (-0.1, 1.9)	-0.1 (-1.4, 1.3)
	DBP	1[a]	7.4 (0.3, 15.0)*	0.1 (-1.2, 1.4)	1.1 (-0.2, 2.3)	-1.5 (-3.5, 0.5)
		2[b]	1.4 (-4.9, 8.1)	0.0 (-1.3, 1.4)	1.1 (-0.1, 2.4)	-0.7 (-2.5, 1.1)
	Hypertension	1[a]	10.1 (-16.4; 45.0)	4.5 (-1.2; 10.4)	5.8 (-0.5; 12.1)	-2.4 (-10.6; 6.6)
		2[b]	-4.8 (-25.9; 22.2)	4.4 (-1.3; 10.4)	5.5 (-0.6; 11.6)	-0.4 (-8.4; 8.2)

**Time-lagged (changes)**	***Pulse pressure***	1[a]	2.7 (-3.2, 8.9)	0.1 (-0.6, 0.8)	0.7 (-0.1, 1.5)	0.2 (-1.0, 1.3)
		2[b]	0.7 (-5.1, 6.9)	0.1 (-0.6, 0.8)	0.4 (-0.5, 1.2)	-0.2 (-1.4, 1.0)
	SBP	1[a]	3.1 (-1.3; 7.7)	0.0 (-0.5; 0.5)	0.6 (-0.1; 1.3)	0.2 (-0.5; 0.9)
		2[b]	1.1 (-3.1; 5.5)	0.1 (-0.4; 0.6)	0.7 (-0.1; 1.4)	0.2 (-0.5; 0.9)
	DBP	1[a]	5.0 (-1.3; 11.6)	0.0 (-0.9; 0.8)	0.8 (-0.2; 1.8)	0.4 (-0.6; 1.5)
		2[b]	1.2 (-4.6; 7.4)	0.1 (-0.8; 0.9)	0.8 (-0.2; 1.9)	0.5 (-0.5; 1.5)
	Hypertension	1[a]	7.3 (-16.9; 38.5)	1.3 (-2.3; 5.1)	3.0 (-0.9; 7.1)	1.8 (-2.7; 6.6)
		2[b]	-2.5 (-23.4; 24.1)	1.6 (-2.0; 5.4)	2.8 (-0.9; 6.7)	1.9 (-2.5; 6.4)

Data show longitudinal regression coefficients (95% CI), indicating difference in biomarker (in %) per 10 mmHg increase in blood pressure variable or between those subjects with vs without hypertension

[a]Model 1, adjusted for sex, age at onset of type 1 diabetes and smoking status (time-independent covariates) and time

[b]Model 2, model 1 further adjusted for BMI, HbA_{1c}, total cholesterol, serum creatinine, urinary AER and insulin dose (time-dependent covariates except in the baseline model where only their baseline levels were considered); associations of pulse pressure also adjusted for mean arterial pressure

*p<0.05

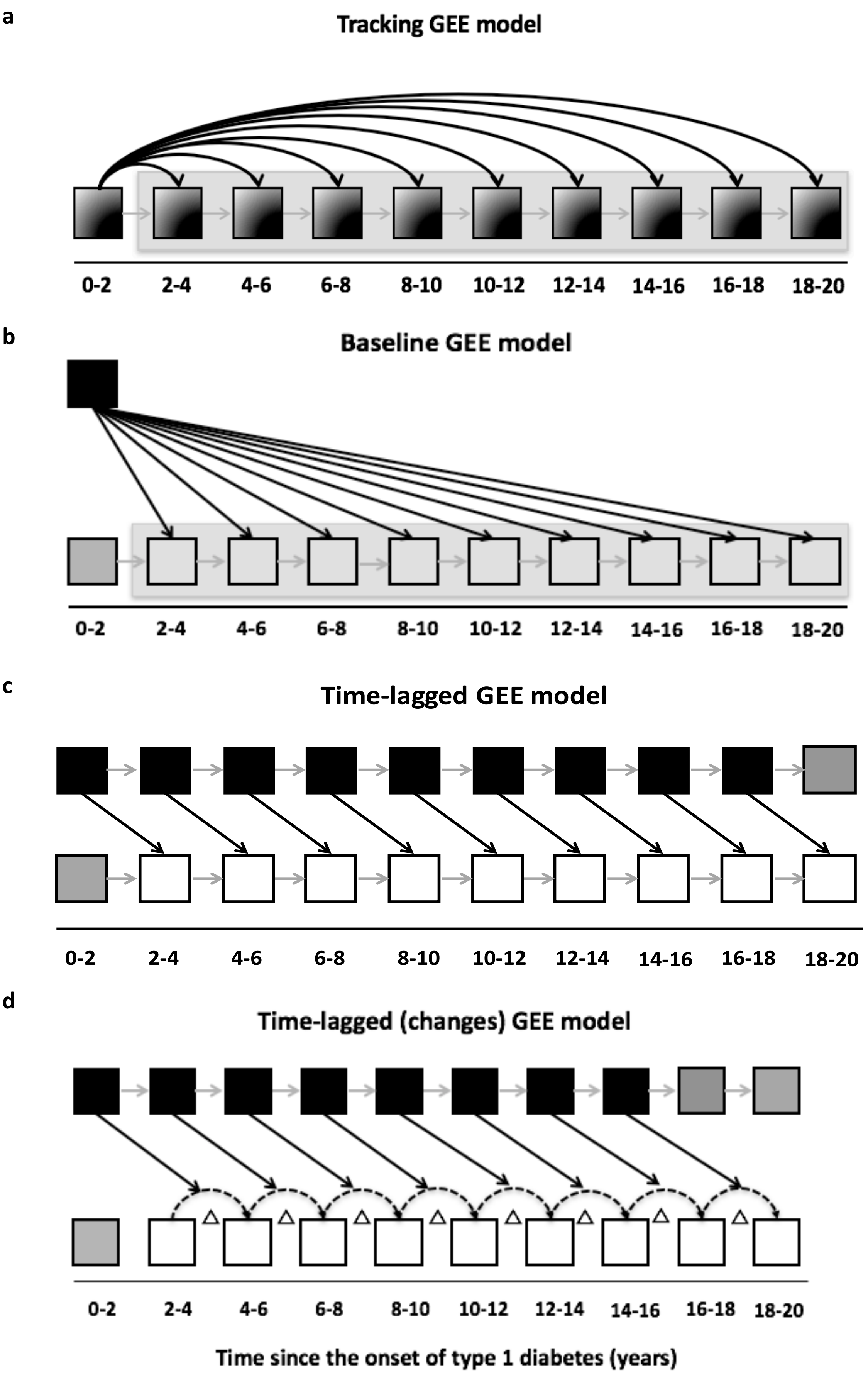
a
Tracking GEE model
0-2
2-4
4-6
6-8
8-10
10-12
12-14
14-16
16-18
18-20
b
Baseline GEE model
0-2
2-4
4-6
6-8
8-10
10-12
12-14
14-16
16-18
18-20
c
Time-lagged GEE model
0-2
2-4
4-6
6-8
8-10
10-12
12-14
14-16
16-18
18-20
d
Time-lagged (changes) GEE model
0-2
2-4
4-6
6-8
8-10
10-12
12-14
14-16
16-18
18-20
Time since the onset of type 1 diabetes (years)

ESM Fig. 1 Illustration of the data aggregation and different models used to analyse the longitudinal data by means of generalised estimating equations (GEE). In tracking analyses (panel a), squares represent main study variables (i.e. biomarkers or blood pressure); in the GEE models illustrated by panels b to d, black squares represent main determinants (i.e. CRP, sICAM-1, sVCAM-1 or sE-selectin) and white squares represent outcome variables (i.e. systolic, diastolic or pulse pressure, or prevalent or incident hypertension); the grey square(s) represent data that, by force of model design, were excluded from the analyses. Note that in models of reverse causation main determinants (black squares) and main outcomes (white squares) were reversed (panes b to d).
